# Topical propranolol for infantile hemangioma: a comprehensive review of mechanisms, clinical efficacy, and novel formulations

**DOI:** 10.3389/fped.2025.1743772

**Published:** 2026-01-06

**Authors:** Feifan Chen, Li Yang, Kunpeng Li, Jiajun Chen, Wenying Liu, Yi Ji, Meng Xia, Jing Xie, Ke Ding, Qiang Zeng, Fang Hou

**Affiliations:** 1 Clinical Medical College, North Sichuan Medical College, Nanchong, Sichuan, China; 2 Department of Pediatric Surgery, Sichuan Provincial People’s Hospital, School of Medicine, University of Electronic Science and Technology of China, Chengdu, China; 3 Southwest Medical University, Luzhou, China; 4 Department of Pediatric Surgery, West China Second University Hospital, Sichuan University, Chengdu, China; 5 Division of Oncology, Department of Pediatric Surgery, West China Hospital of Sichuan University, Chengdu, China

**Keywords:** adverse reactions, efficacy, hemangioma, propranolol, topical application

## Abstract

Infantile hemangioma (IH) is the most common benign soft tissue tumor in children. While most exhibit a tendency for spontaneous regression, a minority can lead to severe, life-threatening complications, necessitating active intervention. Treatment modalities vary depending on the lesion's location, size, and depth. In recent years, topical β-blockers have increasingly become a preferred treatment option for many parents and clinicians. This review aims to systematically summarize the pharmacological mechanisms, clinical efficacy, safety profile, and formulation innovations of topical propranolol in the treatment of IH by synthesizing domestic and international literature, thereby providing a valuable reference for clinical practice and future research.

## 1 Introduction

Infantile hemangioma (IH) is the most prevalent benign soft tissue tumor of infancy, occurring in approximately 4%–10% of full-term infants and up to 8%–12% of preterm infants. Key risk factors include placenta previa, placental insufficiency, decreased gestational age, and very low birth weight (1). About 60% of IH occurs in the head and neck region, some of which can severely affect organ function and aesthetics. Although most IH involutes spontaneously, active pharmacological or surgical intervention is required in cases such as ulceration, bleeding, visual impairment, or airway obstruction, with complications occurring in approximately 10%–15% of all IH cases (2).Traditional IH treatments include corticosteroids, interferon, laser therapy, and surgery, but some modalities are associated with significant side effects and limitations, such as growth retardation and immunosuppression caused by corticosteroids (3). In 2008, Léauté-Labrèze et al. (4) first serendipitously discovered the remarkable efficacy of propranolol (a non-selective β-blocker) against IH, ushering in a new era in the pharmacotherapeutic management of this condition.

With the deepening of research by domestic and international scholars, topical propranolol formulations have gained recognition due to their favorable safety profile, precision targeting, and enhanced convenience for patients and caregivers. These advantages improve treatment compliance and enable for immediate discontinuation of therapy in the evente of adverse reactions (5). Currently, topical propranolol has become an important option for treating superficial IH (6). Compared to oral administration, topical formulations significantly reduce the risk of systemic adverse reactions while maintaining ideal local therapeutic effects, making them particularly suitable for superficial, low-risk lesions (7). Studies worldwide indicate that the efficacy rate of topical propranolol formulations (cream/gel) for superficial IH can reach 78%–90%, with most patients experiencing only mild local adverse reactions and no reported systemic adverse events; in contrast, systemic adverse reactions occurred in 13.7% of patients taking oral propranolol (8). Therefore, by synthesizing domestic and international literature, this review systematically summarizes the pharmacological mechanisms, clinical efficacy, safety profile, and formulation innovations of topical propranolol in the treatment of IH, so as to provide a valuable reference for clinical practice and future research.

## 2 Methods

The literature search for this review was conducted across major English databases (PubMed, Embase, Web of Science, Cochrane Library) along with major Chinese databases [China National Knowledge Infrastructure (CNKI), Wanfang Data, VIP Chinese Science and Technology Periodical Database, SinoMed]. The search was limited to the period from January 2008—coinciding with the seminal report of propranolol's efficacy for IH—up to the present. A systematic search strategy was adopted, which combined Medical Subject Headings (MeSH) and free-text terms, including “infantile hemangioma”, “propranolol”, “topical application”, “pharmacological mechanism”, “clinical efficacy”, “safety” and “dosage form innovation” among others. Boolean operators (AND/OR) were used to construct the search queries. The inclusion criteria were as follows: 1. study population: pediatric patients diagnosed with IH; 2. intervention: focus on treatment with topical propranolol preparations, including studies on pharmacological mechanisms, clinical efficacy, safety, or formulation development; 3. publication type: original research or high-quality reviews published in either Chinese or English. The exclusion criteria comprised: 1. duplicate publications; 2. studies with incomplete, unavailable, or non-extractable data; 3. studies not involving the topical route of administration; 4. preclinical studies not involving animal models of IH or lacking clear translational relevance to clinical practice; and 5. review articles with a methodological quality score below the thresholds set by the Joanna Briggs Institute (JBI) critical appraisal tools.

## 3 Mechanisms of action of propranolol in treating IH

The precise mechanism of propranolol in treating IH has not been fully elucidated. Current evidence suggests it acts through multiple pathways and stages (9).

### 3.1 VEGF signaling pathway inhibition

**Figure 1:**
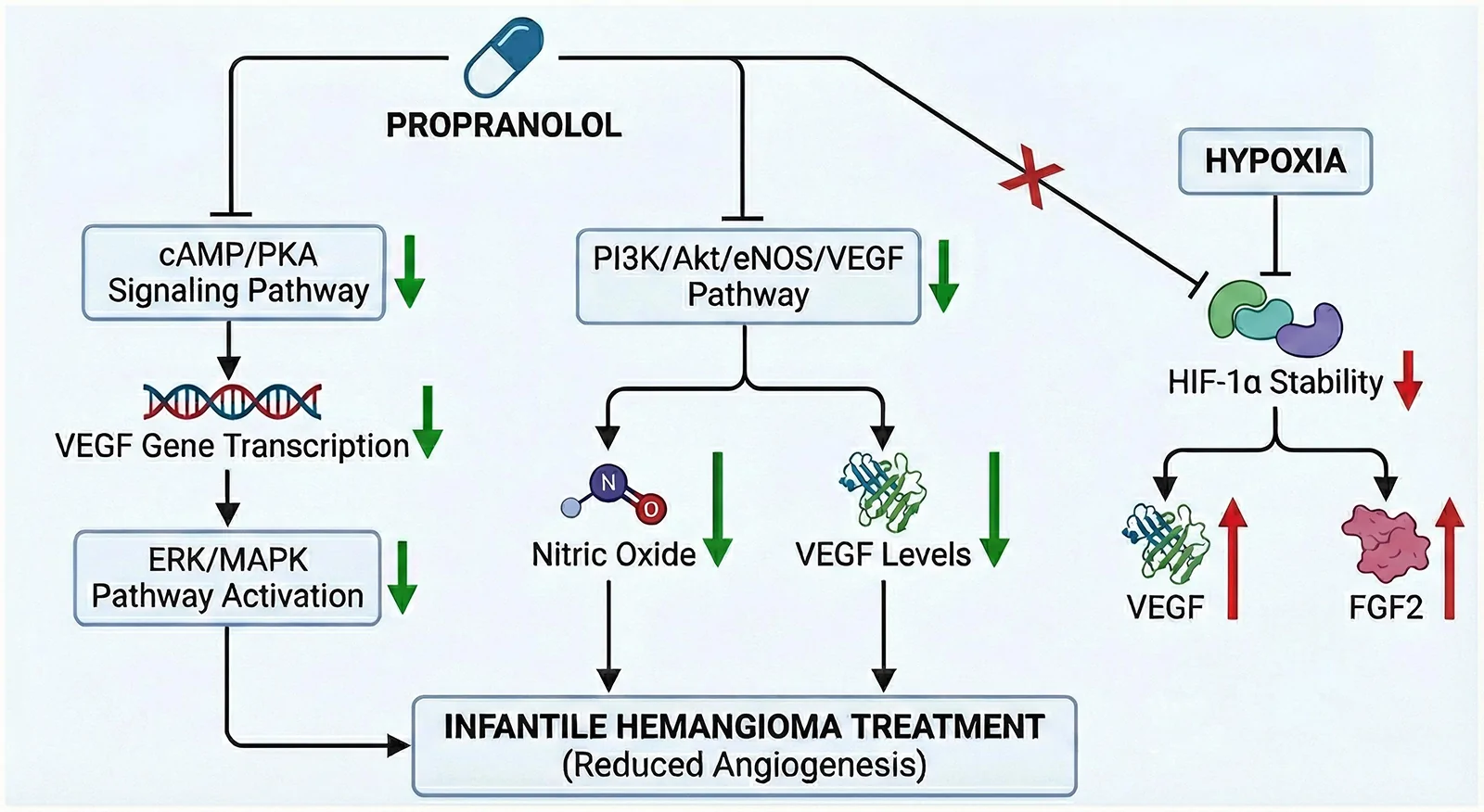
The molecular mechanisms of propranolol by inhibiting angiogenesis in infantile hemangioma. Propranolol inhibits the cAMP/PKA and PI3K/Akt signaling pathways, leading to decreased levels of VEGF (vascular endothelial growth factor) and nitric oxide (represented by green downward arrows). Although hypoxia usually increases HIF-1α stability and promotes growth factors (represented by red upward arrows), propranolol effectively blocks HIF-1α stability, thereby preventing excessive vascular proliferation.

Vascular Endothelial Growth Factor (VEGF) is a key pro-angiogenic factor in IH, showing significantly high expression during the proliferative phase (10). Multiple studies indicate that propranolol inhibits the VEGF signaling pathway at various levels, primarily by modulating downstream signaling molecules (11). For instance, propranolol inhibits the activation of the extracellular signal-regulated kinases (ERK)/mitogen-activated protein kinase (MAPK) pathways via inhibition of cyclic adenosine monophosphate (cAMP)/protein kinase A (PKA) signal transduction, thereby reducing VEGF gene transcription (12). Concurrently, propranolol mediates its effect by suppressing the PI3K/Akt/eNOS/VEGF pathway, resulting in a concomitant reduction in NO and VEGF levels (13) (Figure 1).

Clinical studies confirm that after three months of topical 1% propranolol gel treatment for superficial IH, patients’ plasma VEGF levels decrease significantly (*p* < 0.05), accompanied by marked clinical improvements such as lightening of the IH color and reduction in thickness (14). Notably, propranolol's inhibitory effect on VEGF is tissue-selective, primarily targeting IH endothelial cells with a minimal impact on normal dermal microvasculature (15), this is also an important reason why topical preparations have mild local side effects.

### 3.2 Angiogenesis and regulation

Beyond the VEGF pathway, propranolol modulates the angiogenesis and pathological development of IH through multiple molecular targets. Several studies demonstrate that propranolol significantly reduces the expression level of Hypoxia-Inducible Factor-1α (HIF-1α), a core transcription factor regulating angiogenesis in IH (16). Under hypoxic conditions (common during the rapid proliferative phase of IH), HIF-1α stability increases, subsequently promoting the expression of various pro-angiogenic factors, including VEGF and Fibroblast Growth Factor 2 (FGF2) (17) (Figure 1).

Furthermore, Basic Fibroblast Growth Factor (bFGF) is closely associated with promoting IH endothelial cell proliferation and angiogenesis (18). bFGF is a potent pro-angiogenic factor that acts synergistically with VEGF to promote endothelial cell proliferation and migration (19). Transforming Growth Factor-β (TGF-β) participates in vascular maturation, promoting pericyte differentiation and maintaining vascular stability (20). Propranolol downregulates the expression of bFGF and TGF-β, disrupting the signaling balance of these growth factors and thereby inhibiting IH endothelial cell proliferation and angiogenesis (21).

Recent research has found that propranolol also modulates the Notch signaling pathway, which plays a crucial role in vascular morphogenesis (22). Upon binding of Notch receptors to their ligands, the Notch Intracellular Domain (NICD) is released via γ-secretase-mediated proteolysis, regulating downstream target gene expression (23). The Jagged1/Notch signaling pathway is located downstream of the β2-adrenergic receptor (β2-AR) (24). Studies have indicated that following propranolol treatment, nuclear translocation of the Notch-1 intracellular domain decreases, and the expression of the Delta-like Ligand 4 (DLL4) protein is downregulated, leading to aberrant vascular branching and impeding the maturation of new vessels (25).

In clinical practice, when topical propranolol formulations are administered, these regulatory effects collectively inhibit the expansion of the vascular network in IH. Particularly effective for proliferative IH, they can significantly retard tumor enlargement and lay a solid foundation for subsequent regression.

### 3.3 Renin-angiotensin system regulation

Abnormal activation of the Renin-Angiotensin System (RAS) exists in IH tissue (26), representing another important target for propranolol therapy. Studies have found that plasma renin concentration and Angiotensin II (Ang II) levels are significantly higher in children with IH when compared to those in healthy controls (14). The protein and gene expression levels of RAS pathway components in IH tissue are higher than those in normal skin tissue. This aberrant RAS activation may stem from local tissue hypoxia and increased adrenergic tone. Propranolol may promote IH regression by downregulating the expression of Angiotensin Converting Enzyme (ACE) and Angiotensin II Type 1 Receptor (AT1R) (27).

Topical propranolol also inhibits renin release by blocking β-adrenergic receptors, thereby reducing RAS activity. Mechanistically, Ang II activates the Nicotinamide Adenine Dinucleotide Phosphate (NADPH) oxidase system via the AT1 receptor (28). Following propranolol intervention, significant decreases in plasma renin concentration and Ang II levels are observed after 3 months of treatment (14). This downregulation not only reduces vasoconstriction but also alleviates vascular endothelial oxidative stress injury.

Notably, local RAS components can form an autocrine loop within IH tissue. Angiotensinogen, renin, ACE, and AT1 receptors are co-expressed in IH endothelial cells, establishing a local positive feedback regulation. Propranolol inhibits this loop, reducing AT1 receptor-mediated Protein Kinase Cζ (PKCζ, zeta) signal activation, thereby suppressing VEGF expression and endothelial cell migration (29). This finding explains why the local application of propranolol in clinical practice can effectively control the local growth of IH, even with limited systemic absorption.

### 3.4 Apoptosis, cell cycle regulation, and aquaporin

Another key mechanism by which propranolol promotes IH regression is the induction of endothelial cell apoptosis and cell cycle arrest. Research has shown that by downregulating Ang II expression, propranolol inhibits proliferation and induces apoptosis in human hemangioma endothelial cells (HemECs) via the Protein Kinase B (PKB, also known as AKT) pathway. Additionally, it induces significant alterations in the levels of key cell cycle proteins and cyclin-dependent kinase inhibitors (30). Cell cycle analysis indicates that propranolol arrests endothelial cells in the G0/G1 phase, reducing the proportion of S-phase cells; this arrest is closely associated with the upregulation of cyclin-dependent kinase inhibitors such as p21 and p27 (31). Other studies suggest that the mechanism of β-blockers in treating IH might also involve triggering autophagy in IH-derived endothelial cells (32).

François Moisan et al. (33), through functional protein association network analysis of IH and the knockdown of the Adrenoceptor Beta 2 (ADRB2) gene and Aquaporin 1 (AQP1), demonstrated that propranolol treatment and AQP1 downregulation trigger the same pathways, with AQP1 being a primary driver of the antitumor response to β-blockers. Their functional *in vitro* studies indicated that AQP1-positive Cytotoxic T Lymphocytes (Tc) play a critical role in the IH response to propranolol, and in a Matrigel-based angiogenesis assay, propranolol's regulation of AQP1 in IH-Tc reduced capillary-like tube formation. They concluded that IH sensitivity to propranolol may depend at least partially on crosstalk between lesional vascular cells and stromal Tc. Beyond the aforementioned mechanisms, propranolol's effects also vary dynamically with the treatment stage.

### 3.5 Mechanisms of propranolol action at different stages of IH treatment

The early stage of action (1–3 days post-treatment) is predominantly characterized by vasoconstriction. Propranolol blocks β-adrenergic receptors, inhibiting nitric oxide (NO)-mediated vasodilation signaling, leading to rapid darkening of the lesion surface color and softening of texture. This effect is particularly notable in topical treatment, where transient perilesional vasoconstriction (manifesting as a white halo) is often observed within 30 min of application (34).

The core mechanism in the intermediate stage (weeks) is the inhibition of angiogenesis. Propranolol blocks β-receptors, inhibiting the expression of pro-angiogenic factors such as VEGF, bFGF, and Matrix Metalloproteinases (MMPs), while simultaneously inhibiting the Ras-Raf-MEK-ERK signaling pathway, thereby blocking endothelial cell proliferation (35). This stage is crucial for halting the proliferative growth of the IH.

In the later stage (months), propranolol primarily functions by inducing vascular endothelial cell apoptosis and promoting pericyte differentiation. Studies show that propranolol upregulates the Cysteinyl Aspartate-Specific Proteinase (Caspase) pathway in IH stem cells, triggering apoptosis, while promoting the normalization and maturation of vascular structures (36) (Table 1).

**Table 1:** Multi-stage mechanisms of action of propranolol in infantile hemangioma.

Treatment stage	Early (vasoconstriction)	Intermediate (angiogenesis inhibition)	Late (vascular remodeling)	Sustained effect
Time Frame	1–3 days	Weeks	Months	Long-term post-treatment
Primary Biological Effect	β-blockade → ↓ NO → Vasoconstriction	↓ VEGF/bFGF/MMPs → Angiogenesis Inhibition	Caspase pathway activation → Endothelial cell apoptosis	Pericyte differentiation → Vascular maturation & stabilization
Clinical Manifestations	Darkening, cooling, softening of the lesion	Cessation of growth, volume reduction	Significant shrinkage, structural normalization	Prevention of recurrence, tissue remodeling

The arrow (→) indicates the causal relationship or action pathway between sequential biological processes; the arrow (↓) denotes a decrease in the expression level or activity of relevant factors/indicators.

## 4 Clinical topical propranolol preparations

Oral propranolol faces challenges such as slow absorption, susceptibility to degradation in the gastrointestinal tract, and extensive first-pass metabolism, resulting in low bioavailability (≈30%) and potential systemic side effects. Furthermore, while oral propranolol hydrochloride doses ≥2 mg/kg/day achieve better therapeutic effects, the incidence of systemic adverse events increases significantly, indicating that the cumulative dose of propranolol is a potential risk factor for side effects (37). To circumvent these limitations, topical propranolol formulations have been developed to enhance local efficacy and minimize systemic exposure.

### 4.1 Clinical efficacy evaluation of topical propranolol

The clinical efficacy of topical propranolol is primarily evaluated objectively using the Achauer grading system: Grade I, lesion reduction <25% (Ineffective); Grade II, 26%–50% reduction (Partially Effective); Grade III, 51%–75% reduction (Markedly Effective); Grade IV, >75% reduction (Nearly Resolved). Wang et al. (38) conducted a study involving 40 infants, who were administered 2% propranolol cream topically three times daily. After a 12-month follow-up, therapeutic efficacy was evaluated according to the Achauer criteria. Specifically, 2 cases (5%) achieved Grade Ⅰ, 15 cases (37.5%) achieved Grade Ⅱ, 17 cases (42.5%) achieved Grade Ⅲ, and 6 cases (15%) achieved Grade Ⅳ. The overall marked response rate (Grade III + IV) was 57.5%. Yuan Jin et al. (39) applied topical 1% propranolol hydrochloride cream three times daily to 56 children with IH, observing an effective rate of 91.07% after up to 8 months of treatment. They also pooled 9 studies involving 375 children (aged 30 days to 2 years) with treatment durations of 1–12 months, evaluating the efficacy rate of topical propranolol, with concentrations of cream or gel ranging from 0.5% to 4% across 8 studies. Price et al. (8) analyzed 12 studies on topical propranolol for IH, involving 597 patients aged 3 weeks to 33 months, with treatment periods of 2 weeks to 16.5 months. At the end of treatment, the overall response rate was 90%, and no systemic side effects were reported. A Japanese trial on proliferative IH showed that 5% propranolol cream applied twice daily had an efficacy rate of 68.8% at week 24, with a good safety profile (7). Jacob Mashiah et al. (40) conducted a retrospective study on all IH cases treated with 4% topical propranolol gel between 2013 and 2015. A total of 63 patients with 75 IH were enrolled. Among them, 43 lesions (57.3%) showed a good response to treatment, 19 lesions (25.3%) achieved partial remission, and 13 lesions (17.33%) had poor or no remission. Consequently, 62 lesions (82.6%) exhibited either a good response or partial remission to the treatment. A trial involving 51 children with IH confirmed that after 7 months of propranolol gel treatment, 56.87% of patients achieved > 50% lesion reduction (Grade III + IV); however, the efficacy rate for deep-type IH (43.1%) was significantly lower than that for superficial-type IH (78.6%) (41).

Notably, the efficacy rates of topical propranolol vary considerably across studies (65.5%–91.9%). Although concentration is a key variable influencing efficacy, available data suggest that the relationship is not a simple linear positive correlation. The 1%–3% range appears to be the “high-efficacy concentration range” for topical propranolol, as within which satisfactory therapeutic effects are achieved. A higher concentration of 5% did not enhance efficacy and might even reduce treatment compliance due to increased local irritation.

Besides concentration, the drug formulation is another critical factor affecting the efficacy of topical propranolol. Differences in skin permeability, drug stability, and targeting among formulations directly lead to variations in efficacy even at the same concentration. Among various formulations, gels and creams demonstrate distinct advantages, as their penetration enhancement and stability are better suited for treating superficial IH. They facilitate better penetration into the skin layers, allowing the active drug to reach the lesion site effectively while maintaining drug stability for sustained action. Solution formulations, however, exhibit poor efficacy due to insufficient skin penetration. Even at a 1% concentration within the “high-efficacy range” the efficacy of solutions is significantly lower than that of gels and creams at the same concentration (42). In existing studies, the application frequency for topical propranolol is typically “twice daily” or “three times daily”. However, extensive observation and data analysis indicate that, under otherwise equal conditions, the therapeutic outcome does not differ significantly based on the frequency of application alone. Therefore, when formulating treatment plans, excessive focus on application frequency is unnecessary; greater consideration should be given to other factors with more substantial impacts on efficacy, such as concentration, formulation, and patient age.

The type of IH is a crucial factor when using topical propranolol. Superficial IH, located more superficially, allow easier drug penetration to the lesion site, allowing the full exertion of its inhibitory effects, hence their topical efficacy is higher than that for deep/mixed IHs. However, in deep/mixed IH, the drug struggles to reach all pathological areas during penetration, resulting in efficacy rates generally below 70%. This may also relate to poor skin penetration of certain formulations, necessitating the development of enhanced penetration formulations in the future. Thus, topical propranolol preparations may represent an ideal treatment for small, superficial IH, while mixed or deep IH might still require combination with oral therapy.

### 4.2 Analysis of the limitations of existing research

However, existing relevant studies still have several common limitations that require attention: Firstly, the vast majority are single-center, small-sample (mostly *n* < 100) observational studies or case series, lacking support from multi-center, large-scale randomized controlled trials (RCTs). Some adopt self-controlled or open-label designs, which are prone to selection bias and measurement bias, and the reported high response rates may not reflect the true efficacy in the broader pediatric population. Secondly, there is excessive heterogeneity among studies, with insufficient unified control of key variables. Significant variations exist in formulation types (such as gels, creams, and solutions), drug concentrations (0.5%–5%), and base compositions. Notably, the transdermal absorption efficiency of the drug is highly dependent on formulation technology. Meanwhile, treatment duration (1–16.5 months), administration frequency, timing of efficacy assessment, and evaluation tools (e.g., Achauer four-grade standard, photographic comparison) have not been standardized, making effective meta-analysis of data difficult. Thirdly, Follow-up periods are generally short (mostly 6 months to 1 year), with a lack of long-term follow-up data beyond 2 years. This prevents clarification of the potential impacts of the drug on infant growth and development, long-term skin sequelae, post-treatment recurrence rates, and their associations with risk factors (e.g., the degree of lesion resolution at discontinuation, age at the start of medication). Fourthly, most studies only take “lesion size reduction” as the primary endpoint, paying insufficient attention to quality-of-life indicators that are important to patients and their families. This not only hinders the analysis of specific reasons for efficacy differences and the determination of optimal treatment regimens but also limits the promotion of this topical treatment in primary healthcare institutions.

### 4.3 Safety analysis of topical propranolol

None of the current studies have reported severe systemic adverse reactions in children using topical propranolol formulations. For example, in a study conducted by Schneider et al. (43), among 148 patients with IH who received topical treatment with propranolol gel for 12 weeks, 147 achieved significant improvement, with adverse reactions being rare. Ren et al. (5) provided a detailed comparative analysis, concluding that topical formulations can effectively control sustained drug release, bypass first-pass metabolism, reduce the incidence of systemic adverse reactions, and increase drug concentration at the lesion site. Yan et al. (44) conducted animal studies on the safety of 1% propranolol hydrochloride cream, single and multiple applications caused no observable irritation to normal or damaged skin in New Zealand white rabbits. In guinea pigs, the cream induced mild skin sensitization but no significant systemic adverse reactions. Foreign animal studies also indicate that topical propranolol formulations cause no significant skin irritation or severe systemic reactions in rabbits (45). Although systemic and local adverse reactions are rare when using topical propranolol formulations, the side effects are closely linked to dosage and typically exhibit “dose-dependent” characteristics. Higher doses may increase both the frequency and severity of adverse reactions, making proper dosage control crucial for minimizing risks. However, the larger the superficial IH, the higher the corresponding dosage of topical propranolol will be used. Therefore, determining a safe dosage relative to lesion size still needs further study.

## 5 Development of novel topical formulations

To improve the convenience and compliance of topical treatment, various innovative propranolol formulations have been or are under development, including: Propranolol Hydrochloride Cubic Liquid Crystal Gel (46), which enhances skin permeability and prolongs drug residence time; Propranolol Hydrochloride Thermosensitive Gel (47), which forms a protective film upon skin contact to enable sustained release; and Propranolol-loaded Nanostructured Lipid Carriers (NLCs) (48), which exhibit favorable carrier stability and potent skin penetration. Wu et al. (49) developed a novel propranolol hydrochloride drug delivery system by encapsulating propranolol in Mesoporous Silica Nanoparticles (MSNs). Their research showed that this nano-drug delivery system exhibited not only enhanced skin penetration but also effective inhibition of Hemangioma Stem Cell (Hem-SC) proliferation and suppression of IH growth in a xenograft model. These innovative topical formulations hold promise for improving the convenience and compliance of IH treatment.

Besides the aforementioned propranolol topical formulations, other β-blocker topical formulations are also used for IH. Timolol (50, 51), a potent β-blocker with 8 times the β-blocking potency of propranolol, shows significant efficacy with minimal systemic adverse reactions when applied topically and is currently widely used. Carteolol (52), another potent non-selective β-blocker, has shown efficacy in treating medium-low risk, superficial, and proliferative IH when used topically as a 2% ophthalmic solution, with common adverse reactions including skin eczema and ulceration.

## 6 Conclusion and future directions

This review systematically analyzes the current evidence on topical propranolol for IH, clarifies its core value as a localized therapy, and highlights its key limitations. The main findings indicate that propranolol exerts its therapeutic effects through dynamic, multi-phase mechanisms, with efficacy being highly contingent on lesion morphology. All included studies consistently affirm its favorable local safety profile, with systemic adverse events reported infrequently. These conclusions offer direct guidance for clinical practice: topical propranolol is recommended as a first-line treatment for superficial, low-risk IH. In contrast, deep or high-risk lesions warrant early evaluation for combination therapy, such as concurrent oral propranolol or other interventions.

However, existing studies still have many limitations, therefore, future efforts, besides focusing on mechanistic breakthroughs and optimizing treatment protocols, should prioritize establishing standardized evaluation systems through systematic RCTs. Indicators such as changes in lesion color, changes in lesion volume, vascular density, serum VEGF levels, and plasma drug concentrations should be incorporated. Unified medication and evaluation standards are needed to enhance comparability across studies. Actively developing standardized formulations with defined transdermal absorption efficiency (e.g., controlled-release gels, patches) and establishing individualized dosing regimens based on patient age, weight, and lesion volume are crucial for more effective treatment of superficial IH.

For IH in special locations, such as mucosal (e.g., oral, genital) areas, the development of mucoadhesive formulations warrants future investigation. For special types of IH where monotherapy has limited efficacy, combination therapy research is warranted. For ulcerated or infected IH, combination with local reparative agents could be explored; for deep or rapidly proliferating IH, combination with targeted angiogenesis pathway inhibitors could achieve a “synergistic inhibition” effect by simultaneously blocking β-receptor-mediated pathways and core vascular proliferation pathways. As topical propranolol might cause local skin dryness or irritation, combination with moisturizing and anti-inflammatory agents such as mucopolysaccharide polysulfate cream could potentially reduce the incidence of local adverse reactions without compromising efficacy. Research in these directions holds considerable practical significance for the clinical management of IH.
